# Sleep Disruption Worsens Seizures: Neuroinflammation as a Potential Mechanistic Link

**DOI:** 10.3390/ijms222212531

**Published:** 2021-11-20

**Authors:** Herlinda Bonilla-Jaime, Helena Zeleke, Asheebo Rojas, Claudia Espinosa-Garcia

**Affiliations:** 1 Departamento de Biología de la Reproducción, Área de Biología Conductual y Reproductiva, Universidad Autónoma Metropolitana-Iztapalapa, Ciudad de Mexico CP 09340, Mexico; bjh@xanum.uam.mx; 2 Neuroscience and Behavioral Biology Program, College of Arts and Sciences, Emory University, Atlanta, GA 30322, USA; helena.abebaw.zeleke@emory.edu; 3 Department of Pharmacology and Chemical Biology, School of Medicine, Emory University, Atlanta, GA 30322, USA

**Keywords:** sleep, seizures, epilepsy, neuroinflammation, neurodegeneration

## Abstract

Sleep disturbances, such as insomnia, obstructive sleep apnea, and daytime sleepiness, are common in people diagnosed with epilepsy. These disturbances can be attributed to nocturnal seizures, psychosocial factors, and/or the use of anti-epileptic drugs with sleep-modifying side effects. Epilepsy patients with poor sleep quality have intensified seizure frequency and disease progression compared to their well-rested counterparts. A better understanding of the complex relationship between sleep and epilepsy is needed, since approximately 20% of seizures and more than 90% of sudden unexpected deaths in epilepsy occur during sleep. Emerging studies suggest that neuroinflammation, (e.g., the CNS immune response characterized by the change in expression of inflammatory mediators and glial activation) may be a potential link between sleep deprivation and seizures. Here, we review the mechanisms by which sleep deprivation induces neuroinflammation and propose that neuroinflammation synergizes with seizure activity to worsen neurodegeneration in the epileptic brain. Additionally, we highlight the relevance of sleep interventions, often overlooked by physicians, to manage seizures, prevent epilepsy-related mortality, and improve quality of life.

## 1. Introduction

Sleep is one of the most basic homeostatic processes of human life. It can be defined as a condition of rest with suspended consciousness and is a necessary biological function with numerous restorative effects on brain function. In 2015, the National Sleep Foundation announced 7–9 h as the updated daily sleep duration recommended for healthy adults [1]. For specialized populations, such as the juvenile and elderly, more time in sleep is recommended to maintain normal brain function. The increased sleep recommendation is partly due to a greater “homeostatic sleep drive” which is the force that builds in the body for sleep as the length of time awake increases. The recommendation for sleep time is based on daily uninterrupted sleep. Unfortunately, sleep is often discontinuous due to interruptions. Therefore, sleep quality, a more complex sleep index that accounts for the number of times a person is awoken during rest (sleep depth), as well as a person’s subjective feelings of well-restedness, are also important determinants of how sleep affects health outcomes. Analyses of polysomnography parameters, spectral analytic data, and subjective sleep estimations reveal that sleep depth is naturally reduced in older adults, and is deeper in females than males [2]. However, high inter-individual and intra-individual variation was observed in this study, which suggests the difficulty of stating precise reference values [2]. Nonetheless, consistent deviations from the recommended sleep duration and quality, due to insomnia, sleep apnea, and excessive daytime sleepiness, collectively referred to as “sleep disturbances”, are disproportionately prevalent amongst people with epilepsy (PWE) compared to people without epilepsy.

Epilepsy is a prevalent neurological disorder defined by the presence of spontaneous recurrent seizures (SRS) affecting nearly 50 million people worldwide [3]. Although great strides have been made over the past 20 years in clinical advances in treating epilepsy, it remains the case that one-third of patients cannot be managed by the existing repertoire of drugs and surgery. Furthermore, no effective strategy exists to prevent the development of epilepsy for people at risk. People with epilepsy are burdened by spontaneous seizures, consisting of widespread uncontrolled neuronal activity. Epilepsy can be a debilitating condition not only because of the neuronal damage induced by spontaneous recurrent seizures but also because of the stress that an epilepsy diagnosis can place on a patients’ social and emotional well-being. Due to the stressfulness of the condition, it is often debated when discussing sleep disorders in PWE whether the increase in sleep disturbances is caused by the occurrence of seizures, or whether spontaneous seizures lead to disturbances in sleep patterns. In order to address the heightened risk for PWE to develop sleep irregularities and disorders, the American Epilepsy Association convened the Sleep and Epilepsy Workgroup (SEW) in May of 2019. The SEW consists of sleep and epilepsy medical practitioners and scientists, as well as lay members of the epilepsy community, who collectively work to better understand the root causes and mechanisms of sleep disturbances in PWE. Importantly, members highlighted a need for further elucidation of the interaction between sleep, biological rhythms, and seizures, especially in the context of mortality. For example, Sudden Unexpected Death in Epilepsy (SUDEP), which is the clinical term used to describe “sudden, unexpected, witnessed or unwitnessed, non-traumatic, and non-drowning death that occurs in benign circumstances in an individual with epilepsy” [4], is suspected to be associated with circadian rhythms and sleep [5]. Recent reports from the European Academy of Neurology, the European Sleep Research Society, and the International League Against Epilepsy Europe [6] also emphasize the importance of identifying and treating sleep disorders to control seizure frequency, improve patient quality of life, and decrease mortality in PWE. Thus, it is of great importance to demystify the connection between sleep disturbances and epileptic seizures. A potential common link between sleep disturbances and epilepsy could be neuroinflammation, as both seizures and sleep disruption individually produce a robust neuroinflammatory response in the brain.

Neuroinflammation, which consists of glial activation and proliferation, as well as the change in expression and release of inflammatory mediators after an initial insult to the brain, is a contributing factor to the conversion of a normal brain to an epileptic brain—a process termed epileptogenesis [7]. Although it is widely known that several neuroinflammatory pathways are upregulated in epilepsy, mechanisms for effectively detecting changes in neuroinflammation at the patient bedside are limited. Similarly, pharmacologic agents that directly target the neuroinflammatory component of epileptic seizures are not yet widely applied in the clinic. It is plausible that neuroinflammation caused by sleep disruption synergizes with the underlying neuroinflammatory response induced by seizures to produce a heightened injury in the brain that, in turn, worsens sleep disturbances and epilepsy. Thus, sleep disruption-induced neuroinflammation is a likely irritant to the epileptic brain and should be investigated as a mechanism for, and potential treatment target against, epilepsy progression in PWE.

The current review focuses on the groundbreaking work done to clarify the contribution of sleep disturbances to epilepsy progression, focusing on neuroinflammation as a primary component of this process. Furthermore, multiple lines of evidence are provided supporting the application of anti-inflammatory treatments to combat both sleep disruption-induced and seizure-induced neuroinflammation. Data from both preclinical and clinical studies, as well as data from animal experiments, are discussed. Additionally, medical therapies currently administered for epilepsy and comorbid sleep disturbances are also highlighted.

## 2. Sleep Disturbances in Epilepsy

Sleep is fundamental for good health and wellbeing, however, seizures and the use of various antiepileptic drugs, along with stress, are important factors affecting sleep quality in patients with epilepsy. Stress effects on sleep homeostasis are well-known to result in a reduction of rapid eye movement (REM) sleep, slow-wave sleep (NREM), and sleep efficiency, as well as increased awakenings [8]. Therefore, hyperactivation of the hypothalamic–pituitary–suprarenal axis [9], its deregulation, and, more importantly, alterations in the circadian rhythmicity of cortisol secretion [9,10,11], could underlie some forms of epilepsy and the comorbid deregulation with mood [12]. Together with anxiety, these factors are closely related, leading to a greater frequency of poor sleep quality and insomnia [13]. Furthermore, deregulation of the hypothalamic–pituitary–suprarenal axis causes a deficit in raphe-hippocampal serotonergic transmission, which can cause depression. Hence, alterations in serotoninergic transmission may be a clinical link between epilepsy and insomnia [14]. In addition to depressive mood and anxiety, perceived sleep insufficiency has been found to be strongly linked to a higher frequency of poor sleep quality and insomnia [13].

**Figure 1:**
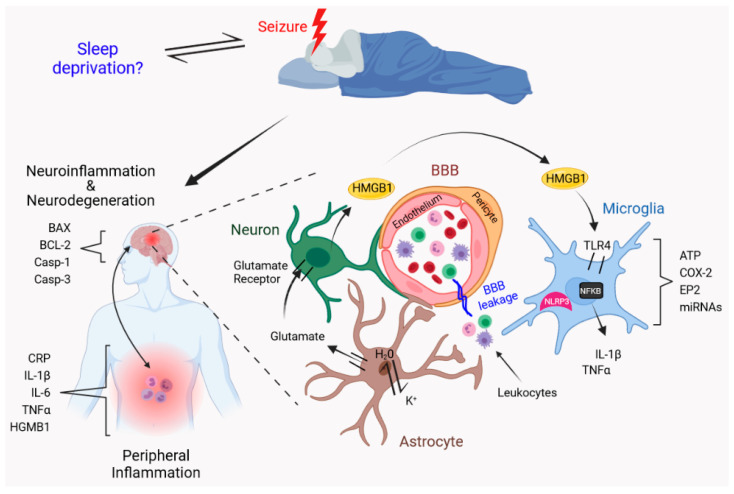
Proposed mechanisms of sleep disruption that contribute to neuroinflammation and worsen neurodegeneration in the epileptic brain. After seizures, peripheral and brain-resident immune cells become active, release danger signals, and produce pro-inflammatory mediators, including cytokines, complement factors, prostaglandins, among others, triggering a sustained vicious circle of neuroinflammation characterized by monocytic infiltration to the brain, astrocytic/microglial activation, and pro-inflammatory cytokine production, which ultimately leads to neurodegeneration. We propose that sleep disruption can act as a second inflammatory hit that increases seizure frequency and aggravates neuronal loss, thus favoring the progression of epilepsy. This figure was created with BioRender.com.

**Figure 2:**
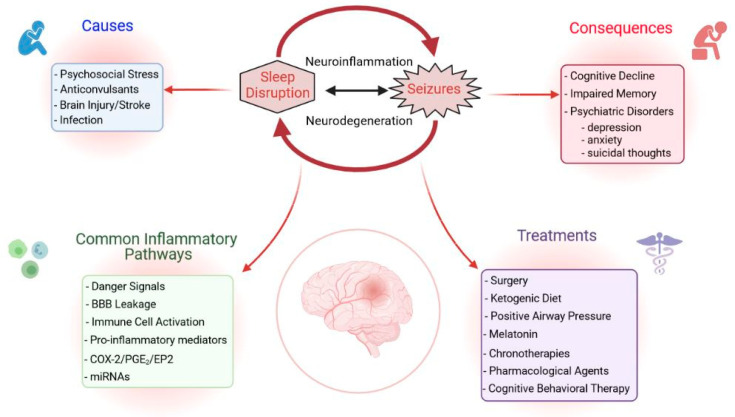
Neuroinflammation as a potential link in the vicious cycle of sleep and epilepsy. This figure was created with BioRender.com.

Sleep disorders negatively impact epilepsy and seizures can also aggravate certain sleep disorders (Figure 1 and Figure 2). This suggests that early diagnosis and treatment of coexisting disorders can help improve a patients’ condition and control of seizures [15]. The many studies on the prevalence of sleep disorders in PWE conducted to date have contradictory results. These differences have been attributed to the diverse groups of patients studied, the types of antiepileptic drugs prescribed, the timing of seizures, the frequency of excessive daytime sleepiness (EDS), the precise syndrome, and the occurrence of nocturnal seizures [16,17,18]. Although data differ among studies, there is no doubt that the comorbidities of epilepsy are diverse and severe. PWE have a high prevalence of sleep disorders, such as insomnia, EDS, obstructive sleep apnea (OSA), restless leg syndrome, and bruxism [11,19]. Any unaddressed comorbidity, regardless of the epileptic patient’s status, can deteriorate her/his quality of life and lead to higher mortality. Each comorbidity requires specialized medical attention, but the experts in the various fields involved may have only limited interaction.

Pre-clinical studies have revealed alterations in sleep patterns marked by a dysfunction in the regulation of sleep-wakefulness cyclicity. The effect of seizures on sleep has been studied in cats and kindled rats. Cohen and Dement [20] found that electrically induced generalized seizures in cats suppressed REM sleep. Raol and Meti [21] also studied the sleep patterns of amygdala kindled rats and found that after the stimulation of five consecutive seizures, rats had a decrease in REM duration, an effect that persisted for 28 days. Other studies in rats have shown that a single seizure, induced by either amygdala or hippocampal kindling, can cause a decrease in REM sleep [22,23]. An animal model using rats genetically predisposed to audiogenic seizures [24] evaluated the effect of seizures on sleep architecture, reporting a prolonged reduction in fast-wave sleep (FWS) concomitant with diminished slow-wave sleep (SWS), with no subsequent compensatory increase in this phase of the sleep–wakefulness cycle [25]. This suggests that generalized paroxysmal attacks provoke disorganization in the functioning of the systems that regulate FWS, though the cerebral synchronization mechanisms in charge of SWS are affected to a lesser extent. This disorganization is what seems to lead to the loss of sleep–wakefulness cyclicity. In an animal model of epilepsy, WAG/Rij rats that displayed SRS were subjected to sleep deprivation for 12 h and investigators observed an increase in sleepiness in the first hours of sleep deprivation with a significant increase in peak wave discharges with greater wakefulness [26]. During the last hours of sleep deprivation, the propensity to sleep increased, inducing the rats to fall asleep more quickly. This reduced the number of spike-wave discharges and as a result the number of peak-wave discharges decreased in the epileptic rats subjected to sleep deprivation.

Despite the fact that epilepsy affects sleep patterns, few clinical and pre-clinical studies have evaluated the effects of lack of sleep on long-term disease outcomes. In humans, sleep deprivation has been used as a procedure for electroencephalography (EEG) activation for clinical diagnoses of anomalies in epilepsy. Comparative studies show that sleep deprivation activates epileptiform discharges, ranging from 23 to 62% in adult patients with definite or suspected seizures [27], because sleep deprivation increases neuronal excitability [28] and precipitate EEG epileptiform discharges [29]. However, a controlled study of patients with refractory partial epilepsy failed to show an effect of sleep deprivation [30]. In this study, patients with refractory partial epilepsy were sleep deprived on alternate nights for 8 days by staying awake between 10 pm and 6 am every other night beginning on Day 2, whereas the control group received 8 h of sleep per night. The results did not show a difference in the number of seizures or seizure latency, suggesting that chronic sleep deprivation likely only increases the risk of seizures.

### 2.1. Insomnia

Insomnia is a sleep disorder in which patients have difficulty in falling or staying asleep. Approximately 30% of adults in Canada complain of symptoms of insomnia and 10% experience insomnia chronically [31,32], while in the USA 27.3% of adults suffer from insomnia [33]. In PWE, the studies indicate a prevalence of insomnia in the range of 24–55% [34,35,36,37,38,39]. For instance, Khatami et al. [34] similarly reported insomnia in 34% of PWE and 28% of controls for the ability to fall asleep, but insomnia associated with sleep maintenance was, in contrast, more common among PWE compared to controls (52% for PWE vs. 38% for controls). In their study, Quigg et al. [38] detected a significant association between the severity of insomnia and certain characteristics of patients with epilepsy, such as younger age, lesser disease duration, use of sleep medication, diagnoses of comorbidities, sleep delay, daytime sleepiness, and depressive states. After controlling for these covariables, the severity of insomnia maintained a strong association with the status of seizures and quality of life. These findings indicate that insomnia is an important comorbidity of epilepsy that is associated with the severity of seizures and poor control, though not all studies relate insomnia to epileptic seizures [36,40]. Other studies, however, have not reported an association between insomnia and the control of seizures [36], and the only variable of epilepsy that increased the probability of moderate or severe insomnia was AED polytherapy, which was considered a marker of drug-resistant epilepsy [40]. Overall, there appears to be a significant relationship between insomnia and poor control of seizures [13,35].

Insomnia can be considered a non-adaptive behavior that results from interactions among the cerebral activities that maintain sleep–wakefulness states. Cerebral activity responding to stressful events can reinforce insomnia. For example, patients report that emotional stress and sleep deprivation are commons triggers of seizures [38,41], and so the hyperactivation hypothesis considers these to be important factors in the development and maintenance of chronic insomnia [9]. Another possible participant in the neurobiology of insomnia is deregulation of the hypothalamic–pituitary–suprarenal axis, specifically alterations in the circadian rhythmicity of cortisol secretion. One consequence of insomnia is sleep deprivation, which could be considered a stressful factor in itself, since excessive wakefulness results in the forced activation of this axis [42], with a positive correlation between insomnia and increased cortisol secretion [43,44]. However, evidence from other studies contradicts this correlation, finding no differences in cortisol levels between insomniacs and control subjects [45]. Therefore, insomnia can occur as a direct consequence of epilepsy itself or be secondary to associated factors, such as depression, the effects of medications, or deregulation of the hypothalamic–pituitary–suprarenal axis [46].

### 2.2. Obstructive Sleep Apnea in Epilepsy

Obstructive sleep apnea (OSA) consists of episodes characterized by the partial (hypopnea) or total (apnea) closing of the upper airways. OSA episodes, which occur during sleep, can interrupt breathing, producing weak gas exchange and oxygen desaturation, with periods of hypoxia and hypercapnia that generally provoke transitory awakenings [47]. Repeated awakenings due to OSA causes significant interruptions and fragmentation of normal sleep that results in sleep deprivation due to prolonged wakefulness or poor sleep quality, but also induces increases in glucose, free fatty acids, and plasma cortisol levels [48] that raise the risk of suffering hypertension and metabolic or inflammatory disorders [49]. This indicates the importance of identifying and treating OSA in PWE, especially since many of these patients cannot initially recognize, or interpret correctly, signs of sleepiness. Therefore, efforts must be made to detect the presence of any underlying respiratory disorder related to sleep, such as OSA. To this end, physicians should ask patients about other symptoms that may be associated with OSA, such as snoring, witnessed apneas, difficulties with memory or concentration, uncontrolled headaches, treatment-resistant hypertension, and fatigue [50].

OSA is a common sleep disorder that has a marked bidirectional comorbidity with epilepsy [51,52,53,54,55]. Although this coexistence has been documented in several studies, the precise pathophysiology of this comorbidity is not yet clearly understood. A meta-analysis of 26 studies revealed a high prevalence of OSA (33.4%) in patients with epilepsy [56]. The prevalence of OSA in adults and children with epilepsy is 40 and 26%, respectively. In relation to the type of seizure, reports suggest an incidence of OSA in 32.2% of PWE that experience focal seizures and 28.2% in patients with generalized seizures [56]. These figures increase in patients with refractory epilepsy. One study of PWE reported a higher prevalence of OSA in patients with refractory epilepsy (43.8% of 32 patients, >1 seizure/month) compared to a group of patients with mild epilepsy (30.7% of 52 patients, 0–1 seizures/month) [57]. Another study reported a higher prevalence of OSA in patients with refractory epilepsy compared to patients with controlled epilepsy [58]. A recent analysis indicates that reduced REM sleep is a pronounced characteristic of both OSA and drug-resistance in PWE, and that treating OSA not only restored REM sleep but produced a concomitant improvement in seizure control [59]. Anticonvulsants, including gabapentin, pregabalin, valproic acid, vigabatrin, and carbamazepine, have been associated with weight gain, which can independently increase the risk of developing OSA [60].

### 2.3. Excessive Daytime Sleepiness in Epilepsy

The second edition of the International Classification of Sleep Disorders (ICSD-3) defines excessive daytime sleepiness (EDS) as “the inability to maintain wakefulness or alertness during the major waking episodes of the day, that results in involuntary lapses of sleepiness or sleep” [61]. Data from some EDS studies suggest a high prevalence of EDS in patients with epilepsy [16,34,35,58,62,63,64,65], although other reports found a similar prevalence to controls (healthy adults) [66,67]. EDS is not classified as a disease or disorder but rather as a symptom that occurs primarily in sleep disorders, such as narcolepsy, OSA, and restless leg syndrome. The second edition of the ICSD states that EDS is interpreted subjectively and may be confounded with tiredness or fatigue, so achieving an accurate diagnosis is challenging. The instrument most often utilized in sleep research to subjectively evaluate ESD is the Epworth Sleepiness Scale (ESS) [68]. This tool can be self-applied quickly using eight typical situations of dozing based on a scale of 0–3 points for each question for a maximum score of 24. For objective evaluations, the multiple sleep latencies test (MSLT) and maintenance of wakefulness tests (MWT) are commonly used.

Some reports indicate ESS scores >10 in 18–47% of patients with epilepsy and 12–17% of controls [16,64,65], with a trend towards higher ESS scores in patients with intractable seizures [58]. However, very few studies analyze EDS subjectively as well as objectively. De Almeida et al. [69] assessed EDS using both tests in 39 patients with temporal lobe epilepsy. The most frequent complaints registered in that study were daytime sleepiness (85%), frequent awakenings (79%), and nocturnal seizures (69%). Of the subjects included in the EDS study, 13% had OSA, and ESS scores correlated with MSLT mean latencies. EDS was found in 36% of participants (ESS score >10) [69]. EDS in PWE can have multifactorial origins [70], often related to lack of sleep subjacent to the effect of medication, alterations of circadian rhythms that interrupt nocturnal sleep, OSA, and restless leg syndrome [18,53,62,71]. The most common medical cause of EDS is OSA, though subjective and objective tests for EDS correlate only weakly with measures of OSA severity [72].

#### Effects of Anti-Epileptic Drugs on Excessive Daytime Sleepiness

Other investigators have interpreted EDS primarily as an adverse effect of treatment with anti-epileptic drugs and frequent seizures [16,17], but the research indicates that the effects of anti-epileptic drugs (AED) on daytime sleepiness depends on the drug prescribed, dosage, and treatment time [35,62,73,74]. Research on the role of AED in EDS show that patients who follow a stable medication regimen do not report more EDS [75,76]. Levetiracetam (Keppra) initially showed an ability to reduce sleep times and alter sleep architecture, but as treatment advanced this effect declined [74]. In the case of a short cycle of monotherapy with topiramate at 200 mg/day, no effect on daytime wakefulness was detected after evaluation with MSLT and an assessment of visual reaction times in 14 patients [77]. Pregabalin improves control of seizures, increases REM sleep, and reduces the N2 state, but also increases ESS scores, though these remain within normal limits, suggesting mild daytime sleepiness [78]. One random, prospective, double-blind study evaluated the effect of pregabalin at 300 mg/day vs. a placebo on polysomnographic variables in 17 patients with well-controlled partial seizures and reports of sleep disorders. This study found that the pregabalin group had improved sleep continuity, fewer awakenings, and enhanced wakefulness times after sleep onset with improvements on their scores for the subjective scales applied [79]. Unfortunately, AEDs, including phenobarbitone, sodium valproate, and levetiracetam, can cause daytime sleepiness.

### 2.4. Animal Studies

Many studies in rats support the notion that sleep alterations induce neuroinflammation. Depending on its duration, sleep deprivation can produce multiple physiological effects, including increased plasma corticosterone levels [80,81,82]. This suggests that paradoxical sleep deprivation (PSD) induces a stress response [83] as well as alterations in inflammatory markers, including IL-1β, IL-6, IL-17, and TNF-α [81,84,85], thus contributing to the neuroinflammatory process at the central level [86,87]. In the rat hippocampus, sleep deprivation for 48 and 72 h increased levels of the proinflammatory cytokines TNF-α, IL-1β, IL-6, and IL-8, but reduced those of the anti-inflammatory cytokines IL-4 and IL-10 [82]. Sleep restriction for 5 days increased levels of IL-1β and TNF-α mRNA in the cortex, hippocampus, and basal forebrain of rats [88]. Similar results have been reported after 21 days of sleep restriction [89]. In addition, sleep deprivation induces an increase in the number of the microglia with larger cell bodies and thickening processes in the hippocampus of rats after 5 days of sleep fragmentation [90], revealing the adoption of a less ramified morphology, suggestive of immunological activation and presynaptic terminal phagocytosis in the cortex of adolescent mice [86]. Evidence from studies of rabbits indicates that 4 h of sleep deprivation induces increases in plasma IL-1β concentrations [91]. Moreover, chronic sleep restriction increases the density of immune-reactive microglia marked with ionized calcium-1 (Iba1) in four of the ten brain regions involved in the regulation of sleep/wakefulness, including the pre-limbic cortex, central amygdala, lateral hypothalamus, and dorsal raphe nucleus [92]. Iba1 is involved in cytoskeletal reorganization and phagocytosis [93] and Iba1 protein levels are upregulated in the microglia in response to pathophysiological stimuli, along with a higher expression of inflammatory markers [94,95]. Thus, the increased Iba1 immunoreactivity observed in response to chronic sleep restriction could suggest an inflammatory response [92].

Reports on microRNA (miRNAs, micro-regulators of gene expression in various cell types and physiological processes, including microglial function) show that miR146a, miR-27a, miR-181c, miR-203, miR-125b, miR-199, and miR-29b are related to the NF-κB pathway during microglia activation [96]. In fact, some miRNA perform an important role in sleep regulation and can be expressed at distinct moments in various brain structures, including the cortical areas that regulate sleep duration [97]. Interestingly, miR146a and miR155 are both modified by the effects of paradoxical sleep deprivation [81]. miR146a participates in microglial activation via NF-κB, while miR155 appears to be involved in neuroinflammation by other mechanisms. These findings suggest that sleep deprivation induces neuroinflammation by activating microglia. It has also been reported that during inflammation, cells in the choroidal plexus release extracellular vesicles into the cerebrospinal fluid that contain four inflammatory miRNA, specifically miR-1a, miR-9, miR146, and miR-155, which are captured by astrocytes and the microglia, and this, in turn, amplifies the inflammatory response [98]. Overall, sleep deprivation induces an increase in cytosine levels, an effect that could be mediated by microglia, wherein some miRNAs may amplify the response and induce microglia Ml activation.

Astrocytes are also influenced by the effects of sleep deprivation. Observations show that most of the excitatory synapses in the frontal cortex of mice are contacted by peripheral astrocytic processes (PAPs). PAPs approach the synaptic cleft and expand after prolonged wakefulness, presumably due to the need to eliminate glutamate and potassium ions. Evidence suggests that astrocytes engulf axonal organelles and synaptic elements even in healthy mice, such that their constitutive phagocytic activity contributes to the cleaning of damaged cell components [99,100], likely in response to the neuronal activity associated with wakefulness [101]. One pathway that mediates astrocytic phagocytosis is through the MERTK receptor [101] by means of the action of its Gas6 ligand (specific growth arrest protein 6). In response to acute sleep deprivation, MERTK and its Gas6 ligand are upregulated in the cerebral cortex [102,103]. These findings suggest that the lack of sleep can trigger astrocytic phagocytosis and cause microglia activation [86].

## 3. Circadian Rhythms in Epilepsy

Circadian rhythms refer to endogenously maintained physiological cycles with a period of approximately 24 h that respond principally to the light–dark cycle. The most obvious circadian rhythm in humans is the sleep–wake cycle. In humans, the circadian system is made up of tissue-specific clocks, controlled by the master clock, the suprachiasmatic nucleus (SCN) of the hypothalamus [104]. The SCN has two molecular actors, CLOCK (circadian locomotor output cycles caput) and BMAL1 (brain and muscle ARNT-like protein1), which activate the expression of the genes Period (Per1–3), Cryptochrome (Cry1/2), and Rev-erb alpha (reverse erythroblastosis virus alpha) during the day. Per/Cry proteins start to accumulate in the cytoplasm and move to the nucleus to inhibit CLOCK and BMAL1 transcriptional activity. The levels of mRNA and proteins within the SCN oscillate with a quasi-24-h period. The SCN controls sleep, alertness, body temperature, neuronal activity, metabolism, hormone levels (i.e., melatonin, cortisol), and other functions in a circadian manner. Sufficient sleep, synchronized to the appropriate circadian phase (or “biological night”) is important for hormonal homeostasis and function. Whereas sleep deprivation and the associated dysregulation of metabolism and energy expenditure has been linked to obesity, hypertension, stress hormone release, and cardiovascular death [105]. Furthermore, the imbalance of an individual’s biological time for optimal sleep caused by neurological disorders, such as epilepsy, produces a broad category of sleep-related dysfunctions, including difficulty falling asleep at night, poor quality of sleep, early awakening, circadian rhythm disorders, and sleep-related respiratory disorders resulting in daytime fatigue [10,106].

Bernard [107] proposed Molecular Oscillations and Rhythmicity of Epilepsy (MORE) as a conceptual framework for studying and understanding the mechanisms underlying the circadian rhythmicity of seizures and their probabilistic nature. Bernard proposed the existence of circadian oscillators which induce time-dependent changes in the expression of genes, proteins, and metabolites in different cells and organs of the body. Circadian oscillators orchestrate and control the rhythmicity of numerous bodily functions, such as eating and sleeping, and the molecular oscillations in one organ can influence the activity of the molecular oscillations in another organ. Although it is not known how seizures start (the causal event), the MORE hypothesis proposes that a threshold must be reached for a transition between normal and epileptic activity. The MORE hypothesis suggests that molecular oscillations change the architecture of neuronal networks in a circadian fashion. The molecular architecture is such that the activity of the neural networks is low in the morning. For instance, in the morning the transcription of NF-kB is high in pyramidal cells with a greater expression of receptors at pyramidal cell synapses and a lower astrocytic production of ATP. On the other hand, in the afternoon NF-kB transcription and receptor expression decreases, while mitochondria produce more ATP in astrocytes resulting in high network activity. With this model, Bernard [107] propose that the determinants of seizure rhythmicity may stem from molecular oscillations in any cell type in any organ and that molecular circadian oscillations bring the neuronal networks of the epileptogenic zone near seizure threshold at specific times. Seizures occur when internal (i.e., neuromodulators) or external (i.e., sleep deprivation, stress) factors drive the network above the firing threshold.

Stress is related to an increase in the frequency of seizures that is correlated with an increase in cortisol levels [108]. Sleep deprivation is considered a stressor, alters the sleep–wake cycle, and induces an increase in plasma levels of cortisol and cytokines. The increase in cortisol, among other factors regulated by the stress response, could possibly drive the network above the threshold. Furthermore, evidence indicates that circadian variation may mediate epileptic excitability in both humans and animals [109], and that CLOCK/BMAL genes either directly or indirectly via their transcription factors BMAL1/CLOCK can influence the expression of other genes that are causally involved in epilepsy (e.g., PAR bZIP transcription factor, genes DBP, TEF, and HLF) [110,111], including NRSF [112], NRF2 [113], CREB [114], and mTOR [115], which display altered expression in epilepsy. In this regard, Li et al. [111] reported that CLOCK protein is substantially reduced in excitatory and inhibitory neurons in epileptic tissue of patients with focal cortical dysplasia and tuberous sclerosis complex. In mice, deletion of CLOCK in excitatory pyramidal neurons but not inhibitory interneurons led to a lowered seizure threshold and overt seizures during sleep [111], with altered electrophysiological properties of neuronal microcircuits similar to epilepsy [116]. Taken together, these studies suggest that epileptic excitability could be a direct consequence of the loss of CLOCK function in cortical neurons.

Immune system changes throughout the day are influenced by both sleep and the circadian clock [117]. Immune cells reach their maximum output early at night and then progressively reduce until their nadir in the morning [118]. Cytokines, including IL-1, IL-6, and TNF, reach maximum levels during the nocturnal period [119,120]. In particular, it has been demonstrated that systemic levels of IL-6 have a biphasic circadian pattern with two zeniths around 5:00 a.m. and 7:00 p.m. and two nadirs at around 8:00 a.m. and 9:00 p.m. [121]. Furthermore, nocturnal sleep is necessary for increasing IL-6 levels, indicating that its circadian pattern reflects the homeostatic impulse to sleep like a somnolence mediator [122]. Similar immune changes occur in growth hormone release, for which levels peak close to the beginning of nocturnal sleep, but contrasting with cortisol, for which levels reach a minimum before sleeping and peak between 7:00 a.m. and 9:00 a.m. Although the purpose of these variations is unknown, sleep deprivation and stress have demonstrated an ability to change cytokine and cortisol release patterns and can significantly affect immune function in humans.

Being able to profile and predict the rhythmicity of seizures as they relate to the sleep–wake cycle can lead to improvements in seizure control and the treatment of sleep-related comorbidities. Studies of the chronobiology of epilepsy have determined that epileptic seizures tend to recur at certain times of day and that the pattern of seizures varies with the pathophysiology of the epileptic syndrome [109,123]. The occurrence of seizures at specific times of the day has been consistently observed for centuries in individuals with epilepsy. Seizures differ in their distribution between sleep and wakefulness depending on the location of the epileptic focus [124]. In the case of the circadian rhythm of the seizures, they depend on the type of epilepsy (generalized or focal) as well as the seizure initiation site (frontal or temporal) [125,126,127]. Patient age and the type of seizure experienced (e.g., tonic, myoclonic, clonic, or hyper-motor) also plays a role in the diurnal seizure pattern [125,126,127,128]. For example, generalized seizures mainly occur during wakefulness and daytime in children and adults, whereas in the case of mesial temporal lobe seizures, studies indicate that the seizures present a bimodal distribution with a first peak in the late afternoon and a second peak in the morning [129,130]. Nocturnal seizures occur at night [131], but frontal lobe seizures are most likely to occur during sleep and in the evening or early morning hours, whereas parietal seizures peak between 4:00 a.m. to 7:00 a.m. and occipital seizures peak between 4:00 p.m. and 7:00 p.m. [130]. Therefore, knowing the time of onset of seizures could be helpful in planning therapy (chronotherapy). For example, Guilhoto et al. [132] administered a higher dose of nocturnal antiepileptic medications for 17 children with nocturnal/morning seizures, resulting in a 50–90% reduction of seizures. Medical experts and practitioners are slightly cautious regarding the use of chronotherapy in epilepsy due to the mixed melatonin effects where some studies suggest an anti-convulsant effect of melatonin administration to PWE and other studies suggest a pro-convulsive effect. Nevertheless, more studies are needed to validate chronotherapeutic approaches to epilepsy.

Neuronal networks generating wakefulness, NREM sleep and REM sleep give rise to different physiological characteristics influencing the likelihood of seizure occurrence [133,134]. NREM sleep is a state of EEG synchronization possibly leading to sleep seizures and interictal epileptiform abnormalities. In contrast, REM sleep is characterized by EEG desynchronization and loss of skeletal muscle tone. Desynchronization of the EEG impedes seizure propagation and the expression of interictal epileptiform discharges (IEDs) during REM sleep and wakefulness. In 1981, Lugaresi and Cirignotta described patients with frequent attacks in light NREM sleep, characterized by violent movements of the limbs, neck and trunk, with dystonic and tonic features [16]. The attacks, called hypnogenic paroxysmal dystonia, were short in duration and lacked epileptiform EEG features but responded to carbamazepine therapy. This activity was also described in children with a pattern of continuous spike waves during NREM sleep called electrical status epilepticus during slow wave sleep [135,136], supporting an epileptic origin within the frontal lobe, named nocturnal frontal lobe epilepsy (NFLE). Seizures in NFLE may also originate from the temporal lobe, insula, and posterior regions [137]. Usually, during NREM sleep, seizures and interictal epileptiform abnormalities are more frequent. It has also been observed that awakenings tend to activate certain types of epilepsies, such as juvenile myoclonic epilepsy, possibly suggesting that hypersynchronization during awakening is a cause of seizures.

On the other hand, Epilepsy on awakening (EA) is characterized by idiopathic generalized tonic-clonic seizures (IGE), related to the age of the patient. IGEs occur mainly on awakening (independent of the time of day). These patients have a second seizure peak (almost at evening) [138]. In these disorders, seizures are more prominent during the first 2 h after awakening. They are idiopathic or hereditary in 84–90% of patients [124,139], usually beginning before the age of 15, and seizure control is common by age 25 [140]. The GTC seizure can be the only symptom experienced by patients or can be combined with other subsyndromes of idiopathic generalized epilepsy in childhood or adolescence. In patients with EA the EEG shows characteristics of idiopathic generalized epilepsy (generalized spike-wave frequent, foca1 abnormalities rare, and photosensitivity increased).

## 4. Sleep Disruption and Neuroinflammation

### 4.1. Sleep Deprivation-Induced Neuroinflammation

The mechanisms that underlie sleep are not restricted to specific neuronal circuits located in specialized neuroanatomical regions but rather appear to involve various cellular and molecular mediators that interact in the brain. In addition to neurons, glial cells are recognized as important contributors to the regulation of sleep–wake cycles, and pioneering studies have revealed a key role for astrocytes and microglia in the homeostasis of sleep [86,141,142,143]. Glia mediators, including adenosine and pro-inflammatory cytokines interleukin (IL) -1β, IL-6, and the tumor necrosis factor alpha (TNFα), are known to directly affect sleep pressure, duration, and intensity [144,145]. Activated microglia have two states: M1 (pro-inflammatory) or M2 (anti-inflammatory), depending on the nature of the inflammatory stimulus. Microglia release cytokines in response to the ATP released by astrocytes and neurons, which acts on the purinergic P2X7 receptors in microglia to induce NREM sleep [146]. We also know that peripheral cytokines released by immune cells can enter the CNS along diverse pathways and act on regions of the brain. For example, the anterior hypothalamus promotes sleep [147,148] under physiological conditions and in response to neuroinflammation and infection [149,150]. In this regard, some studies report that systemic or intracerebroventricular administration of IL-1β and TNF-α increases NREM sleep [151,152], while this stage of sleep is reduced when IL-1β signaling is blocked [153]. In contrast, administering the anti-inflammatory cytokine IL-10 during the light (inactive) sleep phase inhibited NREM sleep in rabbits [154], possibly through its inhibitory effects on IL-1β and TNF-α production in microglia [155]. One proposal is that TNF-α has somnogenic actions, attracts the microglia Ml process to dendrites and synapses, and thus modulates sleep–wake cycles. Other work has demonstrated that TNF-α increases the expression of Homer1a, an immediate early gene, which plays a key role in synaptic plasticity [144] and modulates the sleep–wake cycle by influencing the expression of orexin in neurons. This neuropeptide (also called hypocretin) regulates excitation, wakefulness, and appetite [156]. Adenosine metabolism also appears as an endogenous regulator of sleep–wake cycles and a stabilizer of synaptic activity, as the high expression of adenosine receptors in glial cells [144,145] and the pro-inflammatory cytokines IL-1β, IL-6, and TNF-α present a rhythmic expression that reaches a maximum in the middle of the light sleep phase [144].

Microglia, as the resident immune cells of the brain, are essential for maintaining cerebral homeostasis, especially through phagocytosis of apoptotic cells, cell debris, and less active synapses throughout the life cycle. In cases of illness, trauma, lesions, and inflammation the microglia action is considered the first cerebral response [157]. These immune cells can proliferate, migrate, transform morphologically, and increase their phagocytosis, as well as release of inflammatory mediators, reactive oxygen species (ROS) and/or trophic factors, to promote cerebral immunity in cooperation with astrocytes and peripheral immune cells [158]. Microglia also respond by orchestrating neuroinflammation through interaction with the peripheral immune cells that invade the brain, especially if the blood–brain barrier (BBB) is compromised [86,92,159]. This occurs in conjunction with vascular cells, astrocytes, and neurons [160]. Acute or chronic alteration of sleep can lead to a pro-inflammatory state, even in the absence of an evident infection or lesion [161,162]. In humans and rodents, the lack of sleep can produce an elevated white blood cell count, increase circulating levels of CRP, IL-1β, IL-6, and TNF-α [159,161,162], and increase the permeability of the BBB [159,162]. This increased permeability of the BBB to circulating molecules seems to be due to the interruption between the interaction of brain endothelial cells with pericytes [163] (Figure 1). Pericytes are positioned on the wall of capillary blood vessels and contribute to blood–brain barrier stabilization through the induction of endothelial tight junctions and the establishment of gap junctions with brain endothelial cells [164]. The evidence indicates that a lack of sleep reduces the expression of markers of the pericyte–endothelial cell interaction (e.g., PDGFR-β and connexin) in microvessels, with detachment of the pericytes from the capillary walls located in the cerebral cortex and hippocampus [165], induced by the low degree of inflammation due to lack of sleep. Together, these actions contribute to the neuroinflammatory environment produced by sleep deprivation (Figure 1).

In humans, sleep deprivation due to age, work-related stress, stress caused by social or family obligations, or sleep disorders like narcolepsy, insomnia, and sleep apnea are associated with negative effects on health and a deterioration in cognition [144]. The elderly experience a phenomenon called sleep fragmentation, and some reports indicate that they present a chronic, low-grade inflammatory state [146] expressed as an increase of inflammatory mediators, such as central and peripheral IL-6 [166], with greater production of reactive oxygen and nitrogen species [167] and suppression of anti-inflammatory mediators and antioxidants [166]. A similar syndrome is caused by chronic sleep loss in the young, suggesting that short or insufficient sleep is associated with low-grade inflammation [161,168,169]. Clinical studies of sleep disorders, including narcolepsy (characterized by excessive daytime sleepiness), sleep/wakefulness fragmentation, hallucinations, sleep paralysis, and nocturnal sleep conditions, show that patients with narcolepsy have high levels of IL-6 and TNFα subtly deregulated in plasma and LCR, consistent with markers of microglia reactivity [170,171] and low levels of chemokine receptor type 1 and type 3 (CCR1 and CCR3) derived from the microglia /macrophages in samples of peripheral blood [172,173]. This reduced expression of chemokine receptors could lead to a defect in microglial recognition and phagocytosis of damaged cells, resulting in a delayed resolution of acute inflammation. This suggests that microglia-mediated inflammation could trigger the loss of hypocretin neurons in cases of narcolepsy [174].

Research indicates that people who do shift work or have long work hours experience health risks at different levels, including risks to the immune system [175,176]. According to ICSD-3, the disorder caused by shift work is characterized by insomnia or somnolence [61]. Shift work has been found to induce continuous sleep deprivation, stress, and changes in the natural circadian pattern that can affect an individuals’ immune responses [177,178,179]. However, there are few studies on this topic and the results are contradictory [180,181]. In an early study of this subject, Nakano et al. [177] conducted follow-up on pink- and grey-collar shift workers and daytime shift workers (fixed and rotational to both). The study demonstrated that these shift workers showed reduced mitogen proliferation of T-lymphocytes to phytohemagglutinin-P and concanavalin A, especially those who worked fixed night shifts. Nagai et al. [179] found that shift workers had reduced amounts of natural killer cells (NK), CD16+ and CD56+, and that night workers had an increase in CD3+ and CD4+ in comparisons performed at two moments (day shift and night shift). Natural killer cell activity was associated with an increase in fatigue compared to daytime workers. One cohort study involving over 10,000 workers at companies in Holland showed that shift workers, especially those who worked the night shift, had a higher risk of infections (e.g., colds, flu, gastroenteritis) than those who worked daytime shifts [178].

The role of sleep in modulating immune function in humans has been tested previously by observing the prolonged effects of sleep deprivation on various immune parameters. Findings show that acute sleep deprivation (50–64 h) is associated with a temporary increase in NK activity and higher T-CD4+ lymphocyte, CD8+, monocyte, granulocyte, and NK cell counts [119,182,183,184]. However, in subjects in whom a partial lack of sleep was induced by restricting sleep time to 4 h/night, NK cell activity decreased to an average of 72%, compared to participants who slept throughout the night [185]. Studies of chronic deprivation—the most common form seen in clinical practice—have also shown reductions in the activity of NK CD16+, CD56+, and CD57+ cell counts, and IL-2 levels [185,186,187,188]. It is well known that these lymphocytes participate in innate immunity, are important in defending against viruses and intracellular bacteria, and are involved in the response to tumor cells [189,190]. The reduced functioning of NK cells is also associated with a greater risk of developing cancer [191]. Likewise, restricting sleep to 4 h/night has led to the production of inflammatory cytokines, which play an important role in the development of cardiovascular and metabolic disorders [85].

Experimental studies have evaluated whether sleep loss induces inflammation the following day. One meta-analysis reported that neither total nor partial nocturnal sleep deprivation nor chronic sleep restriction reliably increased IL-6, TNF-α, or CRP—three circulatory markers of inflammation [192]. Those results, however, contrast with evidence from assessments of upstream cellular and genomic inflammatory processes. Indeed, partial night-time sleep deprivation activates inflammatory signaling pathways, such as those involving nuclear factor-κB (NF-κB), activator protein 1 (AP-1), and signal transducer and transcription activator (STAT) family proteins [193,194,195], while also increasing levels of mRNAs that encode pro-inflammatory cytokines, and TLR4-stimulated monocyte production of IL-6 and TNF-α [85,192]. It has been suggested that acute sleep loss or short sleep duration activates inflammatory signaling pathways, though the translation of these molecular signals into increases in inflammatory peptides may not occur until later, or may require a more persistent period of sleep loss [193]. In an effort to elucidate the mechanisms that lie beneath the connection between sleep deprivation, the immune response, and metabolic processes, one epidemiological study in humans identified genes and pathways through microarrangements of the complete genome by experimentally simulating sleep restriction during one work week in which sleep was limited to 4 h/night for five nights [169]. Results indicated that this experimental restriction of sleep altered the expression of genes related to the immunological function, including activation of B lymphocytes, TLR-4, STAT1 cells, and the production of IL-8 via the signaling of NF-kB. Of the ten most down-regulated genes, TBX21 and LGR6 correlated negatively, but TGFBR3 correlated positively with insufficient sleep. Observations of the TBX21 and TGFBR3 transcriptions showed that they are mediators of the immune system, while LGR6 and STX16 have been repeatedly associated with the progression of cancer. Partial sleep restriction affects the regulation of the signaling pathways related to the immune system. Some of these changes seem to be lasting and may explain, at least in part, how prolonged sleep restriction can contribute to pathological states associated with inflammation, such as cardiometabolic diseases [169]. However, additional studies are necessary to better understand this relationship.

### 4.2. Hypoxia Induced Neuroinflammation in Obstructive Sleep Apnea

OSA is characterized by recurrent episodes of pharyngeal collapse during sleep that result in intermittent hypoxia due to repeated sequences of oxygen desaturation–reoxygenation. This condition is recognized as a chronic, low-grade systemic inflammatory disease, particularly due to its hypoxic component. A growing body of evidence shows that hypoxia induces a neuroinflammatory response that has an essential role in neuronal damage. Hypoxia has been associated with an antioxidant imbalance and changes in oxidative mitochondrial phosphorylation, which results in greater amounts of reactive oxygen and nitrogen species (ROS, RONS), NADPH oxidase, inflammation, and secondary oxidative damage to lipids, proteins, and DNA [196,197]. Hypoxia is associated with high plasma levels of CRP and pro-inflammatory factors, such as IL-6 and TNF-α, which could contribute to excessive daytime sleepiness [121]. Indeed, increased levels of cyclooxygenase-2 (COX-2) and TNF-α messenger RNA have been described in the brains of rats and mice exposed to intermittent hypoxia [198,199]. The intermittent hypoxia component of OSA causes low-grade neuroinflammation in the dorsal hippocampus of mice, including early, though transient, cytokine elevations, delayed but long-term microglia Ml changes, and altered cytokine responses to lipopolysaccharide (LPS) inflammatory challenges [200,201]. In addition, significant astrogliosis has been observed in the cortex and hippocampus of rats exposed to intermittent hypoxia. This condition also induces greater permeability of the BBB [202], allowing circulating cytokines, specifically IL-1α, IL-1β, IL-6, and TNF-α, to cross the BBB and enter the brain [203]. Inflammation alters the function of the BBB as cytokines increase the disorganization of the narrow unions and the permeability of the BBB itself, resulting in the activation of glial cells, specifically microglia, towards the M1 phenotype, which produces oxidative stress and the release of pro-inflammatory cytokines (Figure 1) [204]. During microglia Ml activation, various signaling molecules and transcription factors, including HIF-1α, NF-κB, and STAT1, are regulated under hypoxic conditions [205,206]. HIF1α and NF-κB are well-studied transcription factors that are interrelated and organize a complex inflammatory cascade [207,208,209] and induce classic M1 polarization due to exposure to IFN-γ or LPS [95]. Studies using primary microglial cultures under hypoxia have shown that the mouse microglia cell line BV2 results in activation of the HIF1α pathway, which induces the polarization of microglia to the M1 state [206]. Another idea is that the STAT1 signaling pathway is a fundamental transcription factor that regulates the transition of the microglia into the M1 phenotype, and that the oxidative stress that derives from the hypoxic atmosphere contributes to activating STAT1 in the microglia Ml cells. Evidence from BV2 microglia of wild-type mice and null cells of STAT1 cultured under hypoxic conditions shows that silencing of the STAT1 protein affects the expression of iNOS and CD68, revealing the central role of the STAT1 signaling pathway in the microglia Ml activation during hypoxia [210] and in mediating the translocation of NF-kB p65 to the nucleus, thereby inducing inflammatory events in a model of spinal cord injury [211].

OSA induces cognitive deterioration caused mainly by neuroinflammation and oxidative stress triggered by intermittent, chronic hypoxia. Earlier studies demonstrated that mitochondrial reactive oxygen species are fundamental in tissue lesions related to hypoxia and that damage to the mitochondria seems to activate the inflammasome (NLRP3), a multiprotein cytosolic complex formed by the NLRP3 protein, the apoptosis-associated speck-like protein that contains a caspase recruitment domain (ASC) adapter protein and procaspase-1, which permits activation of the pro-inflammatory caspases that transform the interleukin-1beta precursor (pro-IL-1beta) into the active form in response to cellular danger signals [212]. Since the microglia are resident immune cells in the CNS, the IL-1β cytokines processed by caspase-1 could be released by the microglia under pathological conditions, thus aggravating the progression of neuroinflammation and cognitive deterioration [213]. In this regard, NLRP3 inflammasome-mediated activation of the microglia may play a key role in neuroinflammatory conditions and in inhibiting activation of the NLRP3 inflammasome to attenuate neuroinflammation and improve neurological function in the case of a brain lesion [214]. In addition, the deficiency of NLRP3 acts as a protector by improving the selective autophagy of damaged mitochondria because, under conditions like hypoxia, mitochondrial respiration is deficient and causes damage by producing ROS. This is considered a conserved auto-degradation process, negatively regulated by the NLRP3 inflammasome [215]. As a result, deactivation or pharmacological blocking of the NLRP3 gene may be a potential therapeutic strategy for the associated neurocognitive deterioration secondary to OSA.

## 5. Targeting Neuroinflammation in Epilepsy

Growing evidence in human epileptic brain tissue and in animal seizure models indicates that neuroinflammation contributes to the development of epilepsy [7,216,217,218,219,220,221,222]. While acute inflammation can be beneficial and promote neural tissue repair, chronic inflammation is detrimental and induces neurotoxicity and neuronal hyperexcitability [221,223]. Furthermore, seizure activity per se induces brain inflammation [219].

The peripheral immune system plays a similar role in epileptogenesis [221]. However, inconsistent findings have been reported on peripheral (blood, serum, plasma, and CSF) markers of inflammation in PWE. For instance, Wang et al. [224] showed that interictal elevated serum IL-6, IFNγ, IL-17a, IFNλ3, and CSF IL-6, IL-17a, IFNλ3 levels were associated with seizure severity in PWE. In contrast, Alvim et al. [225] found reduced cytokine (IL-2, IL-4, IL-6, IL-10, IL-17, TNF-α, and IFNγ) levels in plasma, while neurotrophic factor (BDNF, NT3, and NGF) levels were elevated in PWE compared to healthy controls. Moreover, Zhong et al. [226] reported elevated CRP levels in peripheral blood from PWE compared to healthy controls. A similar increase in CRP and cytokine plasma levels were observed in pilocarpine-treated rats when compared to a saline group [227,228].

An inflammatory crosstalk exists between the periphery and the brain after seizures (Figure 1) [221]. Riazi et al. [229] and De Caro et al. [230] demonstrated that intestinal inflammation increases CNS excitability and seizure susceptibility in pentylenetetrazol (PTZ)-treated rodents. Similarly, Ho et al. [231] showed that LPS-induced peripheral inflammation worsens hippocampal microglial activation and enhanced pro-inflammatory cytokine production in KA-treated rats.

Multiple signaling pathways play a role in the neuroinflammatory response associated with epilepsy, including danger signals release, pro-inflammatory cytokine production, and glial activation (Figure 1) [7,216,217,218,219,220,221,222].

### 5.1. Danger Signals and Pro-Inflammatory Cytokines

High mobility group box-1 (HMGB1), a ubiquitous nuclear protein, is one of the many danger signals released by immune cells or necrotic cells after brain injuries. Several studies have reported elevated HMGB1 levels in the serum/plasma/CSF [232,233,234,235] and brains [233,236] of epileptic patients or animal seizure models [113,237], correlating with higher risk and severity of epilepsy. Once released into the extracellular space, HMGB1 promotes the recruitment of immune cells to the injury site. HMGB1 binds to its receptors such as toll-like receptor 4 (TLR4), triggering the activation of the nuclear factor kappa-B (NF-κB). The TLR4/NF-κB signaling pathway, in turn, activates the inflammasomes―multiprotein complexes that induce pro-inflammatory cytokine production. Pro-inflammatory cytokines, such as IL-1β, act as neuromodulators by interacting with glutamate receptors, e.g., N-methyl-D-aspartate (NMDA) receptors, leading to neuronal hyperexcitability [238,239,240,241]. In epilepsy patients, HMGB1 is known to mediate microglia activation and pro-inflammatory cytokine production via the TLR4/NF-κB pathway (Figure 1) [233,242].

### 5.2. COX-2/Prostaglandin E2 Signaling Pathway

Besides inflammasome activation and pro-inflammatory cytokine production, seizures induce other inflammatory mediators, such as cyclooxygenase-2 (COX-2). COX-2, a critical mediator of the inflammatory response, is constitutively expressed in hippocampal and cortical neurons. Within hours after status epilepticus (SE) onset, COX-2 is induced in neurons, and its upregulation persists up to several days after SE [243]. COX-2 induction is associated with neurotoxicity and neurodegeneration in epilepsy [244]. This enzyme synthesizes prostaglandins from arachidonic acid, which propagate neuroinflammation [245]. Genetic deletion of COX-2 in forebrain neurons reduced neurodegeneration in the hippocampal CA1 region, pro-inflammatory cytokine expression, gliosis, leukocyte infiltration, and brain blood barrier (BBB) leakage, with no effect on seizure intensity after pilocarpine-induced SE [246]. To date, several selective COX-2 inhibitors have been shown to be neuroprotective for epilepsy management [247]. However, many COX-2 inhibitors produce severe adverse effects in PWE, such as cardiotoxicity, and worsen mortality in animal seizure models [248,249]. Therefore, prostaglandin receptors antagonists emerge as promising therapeutic agents to treat epilepsy [218,223,245]. Prostaglandin E2 (PGE2) is the most abundant, and pharmacological inhibition of its receptor, EP2, using novel selective antagonists has been shown to improve survival, weight recovery, cognitive deficits, and reduced neuroinflammation, gliosis, and neurodegeneration in various animal seizure models [250,251,252,253].

### 5.3. Glia

In addition to danger signals, seizures cause the extracellular release of toxic molecules, such as ATP, reactive oxygen species (ROS), or glutamate, which can also activate microglia. Activated microglia produce pro-inflammatory mediators (e.g., cytokines, complement factors, prostaglandins, among others), which in turn transform resting astrocytes into reactive astrocytes [254]. Activated microglia and reactive astrocytes proliferate, display a reactive phenotype characterized by morphological and biochemical changes, and produce pro-inflammatory mediators (Figure 1). Increased numbers of Iba1-positive microglia/macrophages and GFAP-positive astrocytes have been observed in the hippocampus correlating with severe neuronal loss after SE [255,256,257] and in the brain of PWE [258,259]. Recently, Sano et al. [257] found that microglia are the first to be activated, followed by reactive astrocytes and increased susceptibility to seizures. Moreover, reactive astrocytes contribute to neuronal hyperexcitability not only as active players in the neuroinflammatory response but also through the dynamic regulation of neurotransmission and water/potassium homeostasis [260,261,262]. For instance, chronic seizures dysregulate astrocytic glutamate receptor/transporter expression and function in PWE and animal seizure models (Figure 1) [263]. Astrocyte swelling and edema can often occur and cause the release of neurotoxic molecules, leading to neuronal hyperexcitability, seizure development, and epileptogenesis [260].

Besides their role in neuronal excitability and inflammation, recent studies have shown that glia cells can also be protective [260,264]. On the one hand, Wu et al. [265] demonstrated that microglial depletion in SE mice worsened acute seizures and neurodegeneration, and increased spontaneous recurrent seizures (SRS) frequency. Particularly, the ENIGMA-Epilepsy Working Group showed that transient microglial depletion in the early disease phase (i.e., until day 7 after spontaneous seizure onset) prevented neuronal loss and cortical thinning after SE in mice [259]. Interestingly, blockade of microglial proliferation during the chronic disease phase (i.e., starting day 58 until day 71 post-SE) resulted in SRS reduction [266]. Moreover, Zhao et al. [267] showed that mTOR-deficient microglia exposed to an excitatory injury in vitro lose their proliferative and inflammatory responses, as well as fail to effectively engulf damaged neurons. Additionally, Alam et al. [268] validated that impaired microglial autophagy, which acts downstream of mTOR, leads to increased seizure susceptibility and causes severe seizures. Together, these findings strongly suggest that mTOR signaling in microglia is neuroprotective and antiepileptogenic. On the other hand, Sano et al. [257] showed that pharmacological inhibition of microglial activation prevented subsequent reactive astrocytes, aberrant astrocyte calcium signaling, and enhanced seizure susceptibility. Their findings indicate that the therapeutic target to prevent epilepsy after SE should be shifted from microglia in the early phase to astrocytes in the late phase of the disease. Although the protective/restorative role of reactive astrocytes has been shown after cerebral ischemia and traumatic brain injury [260,269], further studies are needed to determine whether reactive astrocytes promote neuron survival or tissue repair in chronic epilepsy.

### 5.4. BBB Leakage

The BBB, composed of endothelial cells, astrocytes, pericytes, and microglia, acts as a selective barrier between the periphery and the brain [270]. Seizure-induced inflammation causes structural and functional changes in the brain, such as BBB leakage (Figure 1) [218,262,271,272]. BBB leakage results in leukocyte infiltration to the brain and the release of mediators that increase vascular permeability, such as vascular endothelial growth factor and transforming growth factor β (TGF-β) [221]. In vivo and in vitro exposure to albumin, a model of BBB leakage, activates TGF-β receptor in astrocytes, resulting in downregulation of potassium channels and NMDA glutamate transporters, which ultimately leads to epileptiform activity [273]. In human brain pericytes, TGF-β attenuated the expression of key chemokines and adhesion molecules involved in leukocyte infiltration [274,275]. Leukocyte infiltration promotes glial activation and the induction of pro-inflammatory cytokine expression, which exacerbates neuronal damage after pilocarpine-induced SE [276] and in PWE [277]. These findings suggest that blockade of leukocyte infiltration could be a therapeutic strategy to prevent seizure-induced sequelae. Moreover, conditional ablation of EP2 receptors in immune myeloid cells and systemic administration of a novel EP2 receptor antagonist prevented monocyte infiltration and BBB leakage, reduced pro-inflammatory cytokines, accelerated weight regain, and ameliorated behavioral deficits after SE [276,278].

### 5.5. miRNAs

Emerging research suggests an important role for numerous upregulated/downregulated miRNAs in seizure-induced neuronal death/apoptosis, excitatory/inhibitory neurotransmission, and neuroinflammation [235,279]. For example, Aronica et al. [280] observed elevated hippocampal miR-146a levels at 1 week that persisted up to 3–4 months after SE in rats, and in PWE with hippocampal sclerosis, reactive astrocytes were the main source, suggesting that miRNAs can modulate the astrocytic inflammatory response triggered by epilepsy. Additionally, Zhang et al. [281] demonstrated that treatment with a miR-146a antagonist was protective against SE by reducing pro-inflammatory cytokine expression, including IL-1β, IL-6, and TNF-α, through NF-κB activation. Similarly, inhibition of miR-103a prevented astrocytic activation and improved neuronal survival in the hippocampus of SE rats by regulating BDNF expression [282]. Moreover, Lu et al. [283] showed that miR-27a-3p inhibitor alleviated seizures, prevented neuronal apoptosis by increasing anti-apoptotic Bcl2 and decreasing pro-apoptotic Bax and Caspase3 expression, and reduced pro-inflammatory cytokine expression, including IL-1β, IL-6, and TNF-α, in the hippocampus of SE rats. In vitro assays showed that miR-27a-3p inhibitor effects were mediated via mitogen-activated protein kinase 4 [283]. In contrast, Fu et al. [284] found decreased serum/hippocampal miR-34c-5p levels in patients with drug-resistant epilepsy and in drug-resistant SE rats correlating with elevated HMGB1 and IL-1β expression and severe hippocampal neuronal loss. Together, these findings indicate that miRNAs can serve as potential targets for the treatment of epilepsy and as molecular biomarkers of epileptogenesis; however, the delivery and safety of miRNA inhibitors remain challenges [235,279].

Therapies interfering with HMGB1/TLR4, NF-κB, IL-1β, COX-2, PGE2/EP2, and TGF-β signaling pathways, and other inflammatory targets have been proposed to treat epilepsy [7,218,221,254]. Table 1 summarizes studies using drugs with anti-inflammatory effects exhibiting anticonvulsant activity in PWE and in animal seizure models.

## 6. Evidence for Sleep Deprivation-Induced Neuroinflammation in Epileptic Rodents

Multiple studies demonstrate that sleep disturbances activate the immune system. Once activated, the immune system triggers an inflammatory response, which, depending on its magnitude and time course, can induce either a longer sleep duration or a disruption of sleep [147,193]. The relationship between sleep and epilepsy is complex and bidirectional. On the one hand, sleep deprivation is known to exacerbate seizures, and on the other hand, some seizures occur predominantly during sleep. Moreover, antiepileptic drugs can interfere with sleep health causing either excessive daytime sleepiness or worsening seizure frequency. To date, only a few preclinical studies have addressed this complex relationship, therefore, there is an unmet need to elucidate the underlying mechanisms of sleep disturbances as biological drivers that worsen epilepsy outcomes. Besides its behavioral and genetic effects, sleep deprivation provoked an imbalance in pro-oxidant and antioxidants, NOX-2 and eNOS were increased, while catalase was reduced in the epileptic cortex [325]. Furthermore, sleep deprivation synergized with epilepsy to elevate miR-146a expression, an important inflammatory modulator [326]. Compelling evidence indicates that the interleukin-1 receptor (IL-1R) mediates epilepsy-induced sleep disruption in IL-1R deficient mice, which exhibited less NREM sleep when compared to sufficiently rested counterparts [327]. However, inconsistent findings regarding the effects of sleep deprivation on levels of neuroinflammatory markers in the epileptic brain have been reported. For example, Mohammed et al. [328] observed an increase in lipid peroxidation and pro-inflammatory cytokines TNF-α, IL-1β, and IL-6, and a reduction in AchE. In contrast, Aboul et al. [329] reported elevated oxidative stress markers and pro-inflammatory cytokine expression in the hippocampus of paradoxical sleep-deprived rats, whereas TNF-α was reduced in epileptic rats after sleep deprivation. Despite these inconsistent findings about whether sleep deprivation enhances or diminishes inflammatory markers, it is clear that neuroinflammation play a key role in epilepsy outcomes. Interestingly, microglial marker Iba1 was elevated in central autonomic brain regions from epilepsy patients who suffered sudden unexpected death (SUDEP) compared to epileptic and non-epileptic controls. These findings support an important role for microglial activation in SUDEP and might indicate a relevant mechanism underlying cardioregulatory failure during a seizure [330]. Further studies examining the impact of sleep deprivation on microglial activation should be conducted to understand other events that affect the inflammatory environment in the epileptic brain.

## 7. Management of Sleep Disruption in Epilepsy

An early diagnosis and treatment of specific sleep disturbances in PWE are key for improving clinical outcomes and quality of life [6,19]. Here, we summarize the approaches that have been used to improve sleep quality and to manage insomnia, OSA, and excessive day sleepiness in PWE (Figure 2).

### 7.1. Surgery

Surgical treatment (anterior temporal lobectomy) of refractory epilepsy significantly improved sleep quality [331], as well as increased total sleep time and REM sleep by reducing the number of seizures [332]. Similarly, Zansmera et al. [333] demonstrated that epilepsy surgery improved sleep quality, sleep architecture, and OSA, resulting in reduced excessive daytime sleepiness among patients with refractory epilepsy.

### 7.2. Ketogenic Diet

A steady energy level may reduce seizures in some PWE. The ketogenic diet, a dietary treatment consisting of a high fat and low protein/carbohydrate content, has been shown to be beneficial for intractable epilepsy [297,298,299,300,301] through multiple mechanisms of action, including an increase in ketone bodies with anticonvulsant and anti-inflammatory effects [321]. For instance, being on a ketogenic diet decreased sleep and improved sleep quality in children with intractable epilepsy [334]. Moreover, there is an ongoing clinical trial based at the University of Wisconsin, Madison aimed at studying the effects of the ketogenic diet on sleep in adults with epilepsy (NCT04193891).

### 7.3. Positive Airway Pressure

Recent studies have found a positive association between OSA and SUDEP risk in PWE, suggesting that screening symptoms for OSA could reduce epilepsy-related mortality, and lead to more effective treatments [335,336]. Positive airway pressure (PAP) is the gold standard of treatment for OSA in PWE [337]. Numerous studies demonstrate that PAP therapy mitigates OSA symptoms and improves seizure control [56,337,338,339,340,341,342]. Nevertheless, additional studies are necessary to discern the relative efficacy of different forms of PAP (i.e., bilevel PAP, nasal continuous PAP). Furthermore, retrospective studies suggest that tonsillectomy and adenoidectomy are effective surgical treatment options for people with epilepsy and comorbid OSA; however, the general efficacy of this type of treatment for reducing seizure frequency and severity on a large scale is still under investigation, as the efficacy of surgery depends on the severity of sleep apnea [343]. These results strongly emphasize the importance of treating OSA in PWE. Despite clinical evidence favoring its effectiveness in improving OSA and reducing seizures, PAP therapy long-term adherence can be challenging for PWE. For instance, a retrospective study found that PWE were less adherent to PAP therapy than controls with OSA but without epilepsy, and PWE were more likely to have a higher number of residual apneas following PAP [344]. A more recent longitudinal study (follow-up period of 5 years) reported a better PAP adherence in newly diagnosed patients, mostly females, and patients with a lower number of total seizures or lower seizure frequency [345]. However, in some cases patients are unable to tolerate PAP [346] and alternative therapies are needed.

### 7.4. Melatonin

The role of melatonin, a pineal hormone involved in the regulation of the sleep–wake cycle, has been extensively investigated as an alternative treatment for sleep disorders in PWE, especially pediatric patients [347,348]. Unfortunately, findings have been inconsistent and contradictory regarding its effectiveness in increasing sleep efficiency and reducing seizures. In children with intractable epilepsy, administration of melatonin improved bedtime resistance, sleep duration, sleep latency, excessive daytime sleepiness, and sleep apnea [349]. In contrast, an additional study reported that children with epilepsy treated with melatonin showed improvements in diurnal seizure frequency, without changes in sleep parameters [350]. Moreover, Jain et al. [351] observed a decrease in sleep latency after treatment with melatonin without changes in seizure frequency. Melatonin is a pleiotropic agent with anti-inflammatory, antioxidant, and neuroprotective properties, which may explain its mixed effects with an ability to reduce seizure activity in some PWE [352].

### 7.5. Chronotherapies

Chronotherapies and personalization of treatment schedules to accommodate sleep-related epilepsy also provides a useful avenue for personalized seizure prevention [126,353,354]. For example, Guilhoto et al. [132] observed that a higher dose of antiepileptic drugs (AEDs) at night led to seizure freedom in 64.7% (11/17) of patients, and 88.2% (15/17) experienced reduced seizure frequency. Clinical efforts must be done to integrate chronotherapy in comprehensive epilepsy care [353], however, further studies with higher numbers of patients and various types of seizures are needed to get a clearer picture of the beneficial effects of chronotherapy in epilepsy.

### 7.6. Cognitive Behavioral Therapy

Cognitive behavioral therapy (CBT) for insomnia has shown some promising results in PWE [355]. However, inconsistent results suggest that more research is needed to clarify optimal approaches, ideal duration of treatment, time course of potential improvement, and best target population [356]. For example, a randomized controlled trial found significant improvements in sleep quality, insomnia severity, sleep hygiene behavior and sleep onset latency in the treatment group (n = 160) [357]. In this trial, CBT also improved anxiety, depression and quality of patient’s life [357]. In contrast, a recent pilot study reported that CBT failed to improve sleep quality and excessive daytime sleepiness in a total of 11 patients with epilepsy [358]. Additionally, there is an ongoing clinical trial based at the Cleveland Clinic aimed at studying the effects of a computerized CBT on insomnia severity index in adults with epilepsy (NCT03632889). 

### 7.7. Pharmacological Therapies

The effects of various AEDs on sleep efficiency have been previously revised [359,360,361]. Some AEDs might affect sleep quality and architecture, causing sleepiness or drowsiness while others can lead to insomnia [362]. However, the effects of AEDs appear to vary in direction and magnitude between patients [15]. Therefore, it is noteworthy that providers carefully evaluate the impact of AED choice on each patient’s quality of sleep and make adjustments accordingly. Although not currently listed as a therapy to manage sleep disruption in epilepsy, we propose that targeting neuroinflammation with novel anti-inflammatory agents might be an innovative therapy for sleep disruption in PWE (Table 1, Figure 1 and Figure 2).

## 8. Conclusions and Future Directions

Sleep is essential for cleaning the brain and maintaining its normal functioning. People with epilepsy often have a reduced sleep duration and poor sleep quality. Nocturnal seizures, psychosocial stress, and the effects of antiepileptic drugs, are the main underlying causes of sleep disturbances in people with epilepsy. The most common sleep disturbances are insomnia, obstructive sleep apnea, and excessive daytime sleepiness, which negatively impact seizure frequency and quality of life (discussed above). Unfortunately, there are methodological limitations for epilepsy patients in the clinic, including a lack of a detailed medical history, inaccurate tools to diagnose sleep problems, and misinterpretation of apneic events as seizure activity, to name just a few. If untreated, sleep disturbances lead to cognitive decline, impaired memory, and comorbid psychiatric conditions (e.g., depression, anxiety, and thoughts of suicide) that worsen sleep quality and epilepsy [11].

The molecular and cellular mechanisms underlying sleep disturbances in epilepsy include but are not restricted to neuroinflammation. Damaged neurons release danger signals, which trigger peripheral and brain inflammatory responses, promoting loss of BBB integrity, leukocyte infiltration into the brain, microglial and astrocytic activation, and pro-inflammatory mediator production. Numerous clinical studies demonstrate that PWE and comorbid sleep disturbances have higher levels of inflammatory markers, correlating with a higher frequency of seizures as compared to PWE who experience good-quality sleep. There are limited studies combining animal seizure models with sleep deprivation; however, findings showed that sleep deprivation similarly increases seizure frequency and exacerbates neuroinflammation. Therefore, therapeutic agents targeting inflammatory signaling pathways could be promising candidates for disease modification in treating epilepsy and should be further investigated for their efficacy in controlling seizures and sleep disturbances in PWE. The findings summarized in this review suggest that routine assessments of systemic inflammatory markers could be useful for identifying existing sleep disturbances in PWE. Furthermore, additional preclinical and clinical studies are urgently needed to definitively clarify the role neuroinflammation plays in the relationship between sleep disturbances and epilepsy progression. With further investigation of this mechanistic pathway, the field will get closer to achieving the ultimate goal of finding useful disease-modifying agents for patients with epilepsy.

## Figures and Tables

**Table 1:** Drugs with anti-inflammatory effects used in clinical and pre-clinical studies to treat epilepsy.

**(A) Clinical studies**				
**Drug**	**Mechanism/Target**	**Pathology**	**Outcome**	**Ref**
Ibuprofen	COX-2 inhibitor	Febrile seizures	Reduced recurrence of a febrile seizure This study lacks inflammation analysis	[249,285]
Dexamethasone	Glucocorticoid with anti-inflammatory and immunosuppressant properties	Drug-resistant pediatric epilepsy Epileptic encephalopathy with continuous spike-and-wave during sleep	Reduced number of seizures Common side effects: increased body weight, anxiety, and insomnia	[286,287]
Minocycline	Suppresses microglial activation and reduces pro-inflammatory cytokine release	Astrocytoma and drug-resistant epilepsy	Reduced seizure frequency This study lacks inflammation analysis	[288]
Aspirin	COX-2 inhibitor	Partial epilepsy	Reduced seizure frequency This study lacks inflammation analysis	[249,289]
Anakinra	IL-1 receptor agonist	Intractable seizures	Reduced number of seizures Changes in IL-1β/IL-10 ratio produced by peripheral blood monocytes	[290]
Tocilizumab	IL-6 receptor inhibitor	New onset refractory status epilepticus	No recurrence of status epilepticus IL-6 levels were normalized 2 out of 7 patients experienced severe adverse events related to infection	[291]
Cenobamate	Sodium channel inhibitor and positive allosteric modulator of GABA_A_ ion channels	Uncontrolled focal (partial)-onset epilepsy	Reduced seizure frequency This study lacks inflammation analysis	[292,293]
Neurosteroids	Pleiotropic actions, including modulation of neuronal excitability and anti-inflammatory properties	Catamenial epilepsy Tuberous Sclerosis Complex	Progesterone was beneficial in reducing seizures in women with perimenstrual exacerbation; its anti-epileptic effects are mainly mediated by allopregnanolone (ALLO), which interacts with the GABA_A_ receptor Ganaxolone (GNX), a synthetic analog of ALLO, reduced seizure frequency These studies lack inflammation analyses	[294,295,296]
Ketogenic diet	Pleiotropic actions, including modulation of neuronal excitability	Absence epilepsy Drug-resistant epilepsy	Reduced seizures These studies lack inflammation analyses	[297,298,299,300,301]
**(B) Pre-clinical studies**				
Celecoxib	COX-2 inhibitor	Pilocarpine	Delayed latency to seizure onset Prevented neuronal death and microglia activation in the hippocampus Decreased in hippocampal levels of pro-inflammatory cytokines, oxidative stress markers, and suppressed MGB1 translocation	[223,302,303]
Indomethacin	COX-2 inhibitor	Pilocarpine	Decreased IL-1β and TNF-α expression	[304]
Aspirin	COX-2 inhibitor	Pilocarpine	Reduced spontaneous recurrent seizures, memory loss, and aberrant neurogenesis These studies lack inflammation analyses	[249,305,306]
Ibuprofen	COX-2 inhibitor	Pentylenetetrazol	Increased latency to seizure and reduced seizure duration Reduced proliferation of astrocytes by increasing autophagy	[307]
Fingolimod (FTY720)	Immunosuppression via modulation of sphingosine-1-phosphate receptors	Lithium–Pilocarpine Excitotoxicity in vitro Kainic acid	Inhibited neuroinflammation, reduced neuronal loss, activation of microglia and astrocytes, and attenuated spontaneous seizures	[308,309]
Dexamethasone	Glucocorticoid with anti-inflammatory and immunosuppressant properties	Pilocarpine Lithium–Pilocarpine	Reduced SE severity and abolished mortality Decreased number of circulating T-cells Reduced BBB damage Reduced hippocampal inflammatory cytokines, prostaglandin E2, and cyclooxygenases Attenuated astrogliosis markers	[286,310]
TG6-10-1	EP2 receptor antagonist	Organophosphorus-induced SE Kainate	Reduced hippocampal neurodegeneration, blunted the inflammatory cytokine burst, and reduced microglial activation	[252,311]
TG8-260	EP2 receptor antagonist	Pilocarpine	Reduced hippocampal neuroinflammation and gliosis, but no effect on neuronal injury nor BBB breakdown	[253]
Neurosteroids	Pleiotropic actions, including modulation of neuronal excitability and anti-inflammatory properties	Pentylenetetrazol Amygdala kindling Kainic acid	Reduced levels of ALLO in the hippocampus correlates with seizure frequency Exogenous treatment with progesterone, ALLO, and GNX suppressed seizures Progesterone and ALLO inhibit inflammatory signaling pathway TLR4/NFκB and NLRP3 inflammasome activation and pro-inflammatory cytokine production in multiple models of brain injury	[295,312,313,314,315,316,317,318]
Ketogenic diet	Pleiotropic actions, including reduction of reactive oxygen species and neuronal excitability, and enhanced production of high-energy molecules	Excitotoxicity in vitro Lithium–Pilocarpine LPS	Improved neuronal survival in vitro Reduced glutamate and enhanced GABA synthesis in the brain, suppressing seizures Reduced levels of pro-inflammatory cytokines in blood and brain after LPS injection Regulated NF-κB activation and pro-inflammatory gene expression in macrophages and microglia	[297,319,320,321,322,323,324]

## Data Availability

Not applicable.
